# Eco-Friendly Synthesis of Silver Nanoparticles from *Ligustrum ovalifolium* Flower and Their Catalytic Applications

**DOI:** 10.3390/nano15141087

**Published:** 2025-07-14

**Authors:** Thangamani Kaliraja, Reddi Mohan Naidu Kalla, Fatimah Ali M. Al-Zahrani, Surya Veerendra Prabhakar Vattikuti, Jaewoong Lee

**Affiliations:** 1 Department of Electronics and Communication Engineering, Sri Venkateswara College of Engineering, Karakambadi Road, Tirupati 517507, India; 2 Department of Fiber System Engineering, Yeungnam University, Gyeongsan 38541, Republic of Korea; 3 Chemistry Department, Faculty of Science, King Khalid University, P.O. Box 9004, Abha 61413, Saudi Arabia; falzhrani@kku.edu.sa; 4 School of Mechanical Engineering, College of Engineering, Yeungnam University, Gyeongsan 38541, Republic of Korea

**Keywords:** eco-friendly method, silver nanoparticles, dye degradation, *Ligustrum ovalifolium* flower extract

## Abstract

The green-chemical preparation of silver nanoparticles (AgNPs) offers a sustainable and environmentally friendly alternative to conventional synthesis methods, thereby representing a paradigm shift in the field of nanotechnology. The biological synthesis process, which involves the synthesis, characterization, and management of materials, as well as their further development at the nanoscale, is the most economical, environmentally friendly, and rapid synthesis process compared to physical and chemical processes. *Ligustrum ovalifolium* flower extract was used for the preparation of AgNPs. The synthesized AgNPs were examined by using UV–visible spectroscopy, XRD, SEM, and TEM analysis. It indicates that AgNPs were formed in good size. AgNPs were applied as a catalyst for the degradation of pollutants, such as methyl orange, Congo red, and methylene blue, which were degraded within 8–16 min. Additionally, the reduction of para-nitrophenol (PNP) to para-aminophenol (PAP) was achieved within 2 min. This work demonstrates a practical, reproducible, and efficient method for synthesizing cost-effective and stable AgNPs, which serve as active catalysts for the rapid degradation of hazardous organic dyes in an aqueous environment.

## 1. Introduction

The main industries that use colorants and dyestuffs are the textile, paint, paper, pulp, and food sectors [1]. Toxic organic compounds are typically present in dye effluents discharged into water [2]. Common colors found in process industry effluents include methylene red, methyl orange (MO), methyl blue (MB), Congo red (CR), crystal violet (CV), acid blue, rhodamine-B (Rh-B), and others. When dyes build up in the water, light cannot reach the surface, which interferes with plant photosynthesis [3]. Aquatic animals and plants perish as a result of the water’s oxygen concentration being reduced. Most contaminants and dyes are complex, non-biodegradable, and extremely difficult to break down. Numerous techniques, including ion exchange processes, coagulation, physisorption, electrochemical precipitation, ultrafiltration, and adsorption, can be used to treat contaminated water [4,5]. Owing to their slow procedure, insufficient pollution removal, high running expenses, etc., the conventional technologies are not practical. Thus, to preserve a healthy ecosystem, organic contaminants and industrial effluents must be effectively and thoroughly removed from water bodies. Moreover, their accumulation in the environment poses serious ecological threats and has been associated with adverse health effects in humans, including mutagenic, carcinogenic, and organ-specific disorders [6].

Nanotechnology is an advanced interdisciplinary field dedicated to the design, synthesis, characterization, and manipulation of materials at the nanoscale, typically ranging from 1 to 100 nm. At this scale, at least one dimension of the material qualifies as a nanoparticle (NP), exhibiting unique physicochemical properties that differ significantly from those of their bulk counterparts. These tailored properties enable nanomaterials to be effectively utilized in a wide array of high-performance and critical applications across various scientific and industrial purposes [7]. The physicochemical characteristics, small size, and high monodispersity of metallic nanoparticles (MNPs) have garnered significant attention in recent years owing to significant catalytic activity, thermal conductivity, light absorption, wettability, and isolation without scattering, subsequently improving performance compared to their bulk counterparts [8,9]. The usage of MNPs in horticulture and biology has been the subject of recent research [10,11]. This is because of their broad antifungal and antibacterial properties against a variety of pathogens, prolonged activity, low toxicity, and potent anti-phytopathogenic fungal effects. Compared to other MNPs, AgNPs have been shown to have strong cytotoxic action against a wide variety of bacteria, fungi, and antioxidant agents [12,13,14].

There are two main methods for the synthesis of AgNPs, which are the physical and chemical methods. These methods are toxic to the environment, expensive, and take a long time for the formation of nanoparticles [15,16]. Owing to these problems, the recently developed eco-friendly method is most suitable for the synthesis of various nanoparticles in an effective way. Some of the eco-friendly materials include yeast and algae [17,18,19,20]. There are various biological methods available in the literature for the synthesis of AgNPs by using various leaf, flower, and fruit extracts of plants such as *Citrus limetta*, *Phaseolus vulgaris*, *Piper chaba*, *Convolvu lus arvensis*, and *Thymbra spicata*, [21,22,23,24,25]. These methods have some drawbacks, such as using strong reducing agents, heating, and additional methods (ultrasonication and microwave) for the formation of AgNPs. Owing to this, there is still a significant need for an eco-friendly method for the synthesis of AgNPs. Hence, we used *Ligustrum lucidum* W.T. Aiton flowers extract for the synthesis of AgNPs.

The beautiful semi-evergreen shrub *Ligustrum ovalifolium* L., sometimes known as California privet or garden privet, is native to East Asia and is frequently grown as a decorative plant. Fruits of the *Ligustrum* (privet) plant are known to contain triterpenoids, flavonoids, and phenolic acids, which give them their immunomodulatory, antihyperglycemic, and anticarcinogenic properties. Recently, synthesis of AgNPs and gold nanoparticles by using fruit extract of *L. ovalifolium* has been applied for the conversion of *para*-nitrophenol to *para*-aminophenol and biological properties [26]. There have been reports on the synthesis of AgNPs using *L. ovalifolium* fruit extract and assessments of their cytotoxic effects on ovarian cancer cells [27]. However, as far as we are aware, there hasn’t been much research carried out on the synthesis of AgNPs utilizing *L. lucidum* flower extract. Thus, the current work was designed to create AgNPs using *L. lucidum* flower extract and investigate their potential as remediation agents (bio-adsorbent) for the reduction of dyes from polluted water, and conversion of para-nitrophenol (PNP) to para-aminophenol (PAP).

## 2. Experimental

### 2.1. Materials

Silver nitrate (AgNO_3_) and NaBH_4_ was purchased from TCI, Tokyo, Japan. Methyl orange (MO), Congo red (CR), methylene blue (MB), ammonium hydroxide (NH_4_OH), and para-nitrophenol were purchased from Daegung Chemicals Co. Ltd, Daejeon, Republic of Korea, and the solutions were prepared using distilled water.

### 2.2. Preparation of Extract

**Figure 1:**
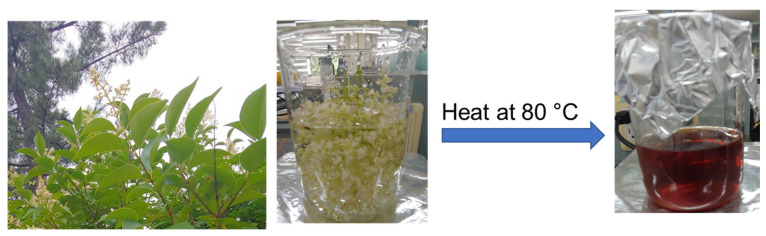
Schematic representation of *L. ovalifolium* flower extract preparation.

The *L. ovalifolium* flowers were flocked and washed 2 times with distilled water; after that, 250 mL of distilled water was poured into a 1 L beaker and boiled at 80 °C for 90 min. After that, the extract was filtered by Whatman filter paper, and the obtained *L. ovalifolium* flower extract was stored at 10 °C in a refrigerator for further use (Figure 1).

### 2.3. Eco-Friendly Synthesis of AgNPs

**Figure 2:**
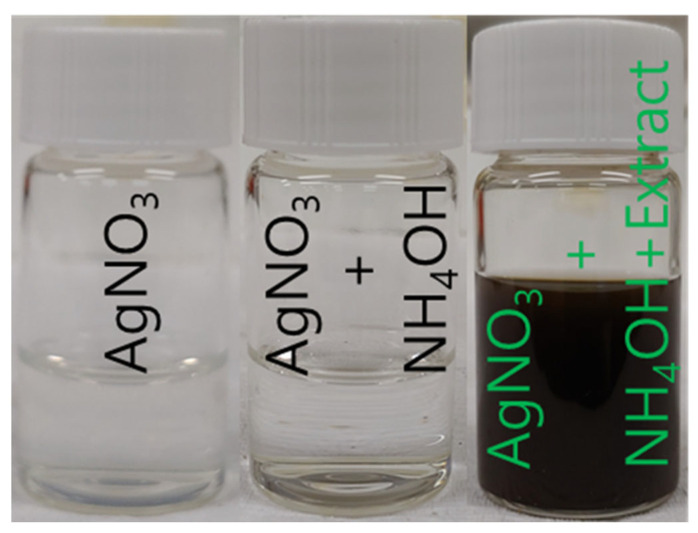
Preparation of AgNPs from *L. ovalifolium* flower extract.

An amount of 10 mL of silver nitrate solution (30 mmol) was mixed with 0.2 mL of ammonium hydroxide, and 5 mL of *L. ovalifolium* flower extract was mixed. Suddenly, the color of the solution changed from white to dark brown, indicating the formation of AgNPs, and was stirred for 1 h Figure 2. The AgNPs were separated by using a centrifuge, Geyongsan, Republic of Korea, washed with water three times, and dried at 80 °C for 8 h. The obtained AgNPs were used as a catalytic application for nitrophenol to aminophenol and various dye degradation studies.

### 2.4. Nitrophenol to Aminophenol

Bio-synthesized AgNPs were employed as a catalyst for the reduction of para-nitrophenol in the presence of NaBH_4_. *p*-nitrophenol (PNP) (1 mmol) was dissolved in 1.85 mL of freshly produced ultra-pure water, and it was combined with 0.15 mL of NaBH_4_ (0.01 mmol). The mixture turned to a deep yellow color, which means the PNP turned to *p*-nitrophenolate ion. The cuvette was transferred to 0.5 and 1 mg of the catalyst, and the UV–vis spectra was measured at different time intervals. This clearly showed the formation of an amine peak in the UV spectra.

### 2.5. Common Method for the Decrease in Organic Dyes

A total of 9.75 mL of MO dye with 50 ppm is added into the beaker; 0.25 mL of NaBH_4_ (0.08 M) is added to the MO dye, and after that 1 mg of AgNPs is added to this beaker and stands at room temperature. The dye sample is taken at different time intervals and checked by a UV–vis absorption spectrophotometer. It indicates the decrease in peak intensities of the dye sample, and the colored solution turns colorless, indicating that the reaction has occurred. The same procedure is adopted for other dyes such as CR and MB.

## 3. Results and Discussion

### 3.1. Characterization of AgNPs

**Figure 3:**
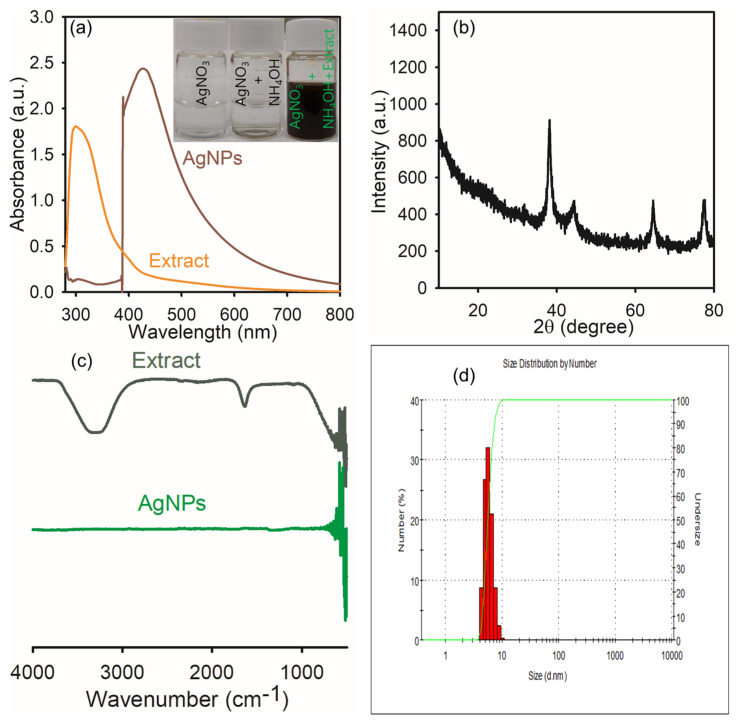
(**a**) UV−visible spectra; (**b**) XRD; (**c**) FT−IR spectra; (**d**) DLS spectra of green chemical synthesis of AgNPs.

Figure 3a clearly indicates the reduction of silver ions and the formation of AgNPs; the color of the samples changes from white to dark brown, which indicates the reduction reaction has occurred and the formation of AgNPs. The UV–visible spectrometer (Geyongsan, Republic of Korea) showed the absorption peak at 432 nm, which indicates that AgNPs were formed by using the *L. ovalifolium* flower extract. The flower extract peak was observed at 306 nm, which indicates that it contains some phytochemical compounds that were reduced by the silver nitrate solution to silver ions. Furthermore, the FT-IR spectra (Geyongsan, Republic of Korea) (Figure 3c) show a peak at 3302 cm^−1^, clearly indicating the presence of -OH functionality, and another peak at 1652 cm^−1^ designates that the carbonyl group forms carboxylic acid functionality in triterpenoids extracted from *Ligustrum lucidum W.T.Aiton* fruits [28]. Additionally, the XRD (Figure 3b) analysis shows crystalline material phase and structural characters. The AgNPs showed Miller indices on the (111), (200), (220), and (311) exteriors, indicating the face-centered cubic assembly of AgNPs with respective angles of 38.09, 44.49, 64.35, and 77.65 degrees. The obtained peaks were exactly matched with the reported data, and the extract reduced AgNO_3_ to AgNPs and stabilized the prepared silver nanoparticles. Synthesized AgNPs using medicinal plants such as *Haplophyllum robustum Bge* [10] have shown strong, similar peaks for AgNPs in the expected region. To ascertain the dispersion of particles in liquids and suspensions, dynamic light scattering is a quick and non-destructive physical technique. Figure 3d demonstrates that the average diameter of the hydrodynamic dimension of the biosynthesized AgNPs derived from *L. ovalifolium* flowers is 10 nm for the concentration of AgNO_3_ (30 mM). The value of PDI = 0.25 determined that the formed particles are single and dispersed. The dispersion of biosynthesized single nanoparticles and the spherical shape of AgNPs can also be seen by the peak created in DLS analysis.

**Figure 4:**
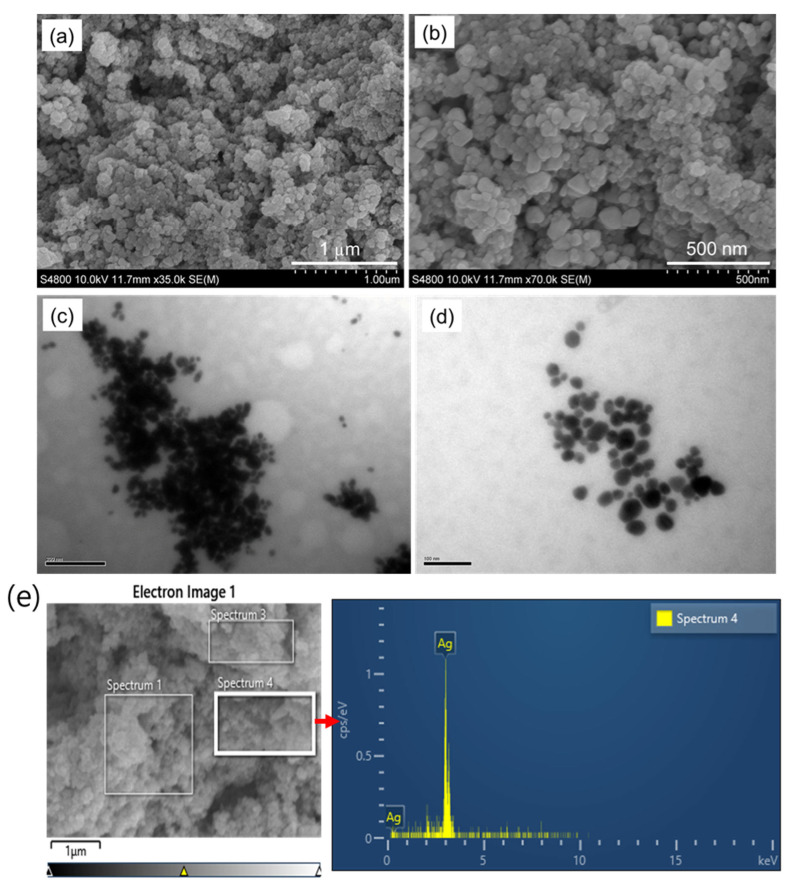
(**a**,**b**) SEM; (**c**,**d**) TEM images of AgNPs; (**e**) SEM-EXD spectrum of AgNPs.

Furthermore, the surface morphology of AgNPs prepared by using *L. ovalifolium* flower extract was studied by scanning electron microscope (SEM) images, which shows that the green chemical synthesized AgNPs are spherical and homogeneous with an average diameter of 50–100 nm (Figure 4a,b). The TEM images depicted in Figure 4c,d indicate that the exact size of AgNPs gained from the *L. ovalifolium* flower has an average diameter less than 100 nm; similar results for the biosynthesis of AgNPs have been described before [11,29]. The elemental analysis was performed using energy-dispersive X-ray spectroscopy, as shown in Figure 4e. The EDX spectrum displays strong and distinct peaks corresponding to silver, specifically at approximately 3 keV, which is characteristic of the silver Lα emission line. This confirms the successful formation and presence of elemental silver in the synthesized nanomaterials. The absence of significant peaks from other elements suggests high purity of the AgNPs. It indicates that the synthesis processes efficiently reduced Ag^+^ ions without the incorporation of major impurities.

### 3.2. Catalytic Reduction of Dyes

Sulfated dyes like methyl orange (MO) and Congo red (CR) are hazardous waste products from the dye industry. On the other hand, methylene blue (MB) is a textile industry effluent that is challenging to remove because it has polar groups and aromatic rings that prevent it from degrading. Hence, we focused on degrading the products by using reducing agents such as hydrazine hydrate or NaBH_4_. The reducing agents themselves cannot reduce or degrade the dyes; they require metal catalysts [1]. Owing to this, we have used the green chemical synthesis of AgNPs for the reduction of MO, CR, and MB. The azo and diazo groups in the MO and CR are the most straightforward methods for breaking down these colored pollutants. In the reduction of the central ring in the MB case, the azo compound acquires hydrogen from NaBH_4_ to form a colorless product.

### 3.3. Catalytic Reduction of MO Dye

**Figure 5:**
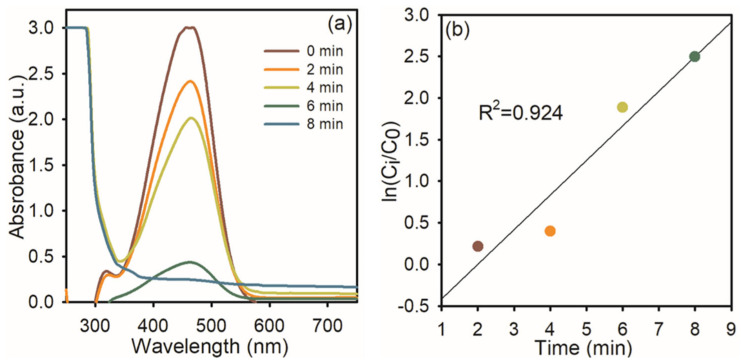
UV−visible spectra of MO dye reduction: (**a**) in the presence of NaBH_4_ and AgNPs; (**b**) the plot of ln (C_i_/C_0_) *Vs* reaction time.

At first, we used NaBH_4_ itself as a catalyst for the degradation of MO dye; it does not change the color of the solution, indicating that NaBH_4_ itself cannot reduce the azo bond in MO. To this same solution, 1 mg of AgNPs was added. The intensity of the absorption peak at 464 nm gradually diminished (Figure 5a), and the orange color of the solution became colorless. This indicates that AgNPs were effectively reduced to the azo bond in MO and diminished effectively up to 92%. Figure 3b shows the linear relationship between ln (C_0_/C_t_) and reaction time, which was used to track the reduction reaction progress, representing the linear association, and it displayed pseudo-first-order kinetics at room temperature, and the rate constant was 0.8344.

### 3.4. Catalytic Reduction of CR Dye

**Figure 6:**
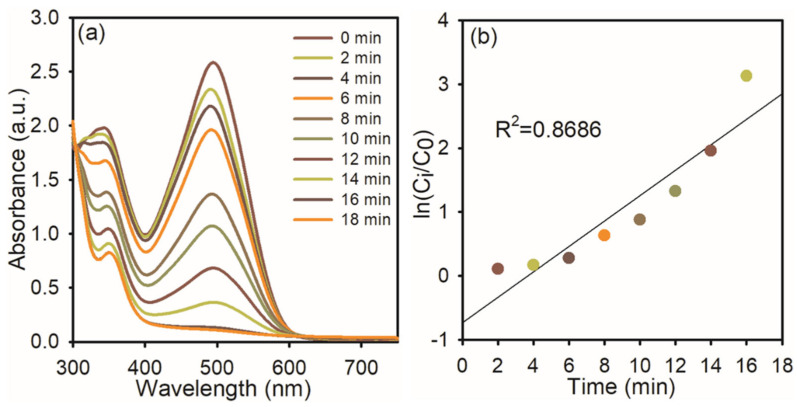
UV−visible spectra of CR dye reduction: (**a**) in the presence of NaBH_4_ and AgNPs; (**b**) the plot of ln (C_i_/C_0_) *Vs* reaction time.

Congo red has a variety of uses, such as staining tissues for microscopic inspection to recognize amyloid deposits and as a gelling agent for poly (vinyl alcohol), and also has potential as a cell wall-preventing agent in fungi, etc. Because CR dissolves in water, it can harm aquatic habitats and create mutagenic, cancer-causing, and reproductive problems in people and other creatures [30]. The synthesized AgNPs were used to examine the catalytic degradation of CR dye in the presence of NaBH_4_ in Figure 6a. An absorption peak at 496 nm was observed in the CR reduction, as found in the UV–visible spectra. The CR dye gradually decreases over time, indicating the progressive degradation of the dye. The reduction in absorbance intensity suggests that the AgNPs effectively catalyze the electron transfer from NaBH_4_ to the dye molecule. The reaction reaches near-completion within 16 min, achieving approximately 96% degradation, and the reaction time (Figure 6b) was determined using ln (C_i_/C_0_), which indicates pseudo-first-order kinetics with a correlation coefficient of 0.8686, supporting the applicability of this model. The calculated rate constant (k) for the reduction processes is approximately 0.8686 min^−1^, indicating a rapid catalytic reaction facilitated by the high surface reactivity of the AgNPs.

### 3.5. Catalytic Reduction of MB Dye

**Figure 7:**
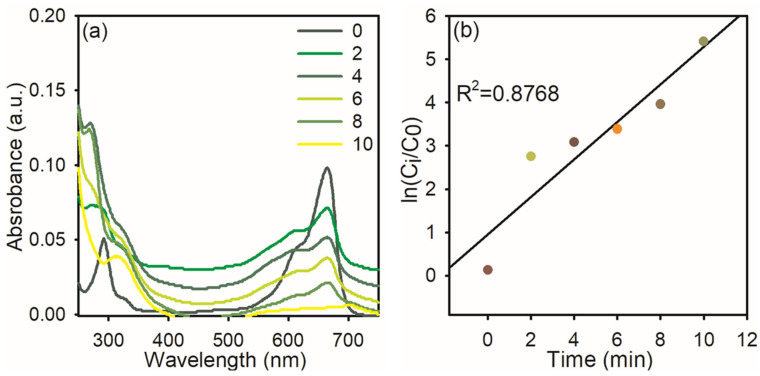
UV–visible spectra of MB dye reduction: (**a**) in the presence of NaBH_4_ and AgNPs; (**b**) the plot of ln (Ci/C0) *Vs* reaction time.

The textile industry typically releases a lot of MB dyes into natural water sources, endangering both human and microbiological health. The significant toxicity of MB dye makes it hazardous to human health above a specific threshold. MB poses a major risk to human health and can have detrimental consequences on the environment because it is poisonous, carcinogenic, and non-biodegradable. MB puts human health at risk for eyesight, digestive and mental issues, respiratory distress, and stomach problems. Additionally, it causes gastritis, jaundice, methemoglobinemia, tissue necrosis, nausea, vomiting, diarrhea, cyanosis, shock, and an elevated heart rate, which results in premature tissue cell death and skin/eye irritations. Because of this, the degradation of MB dye is most significant in environmental research [31]. Hence, we used synthesized AgNPs for the degradation of MB dye in the presence of NaBH_4_. The AgNPs effectively reduced the MB dye within 10 min by 95%, and this followed pseudo-first-order kinetics with a rate constant of 0.876. The obtained results are presented in Figure 7.

The percentage of degradation of the dyes, which reveals that over 91% was degraded, was noted.

**Figure 8:**
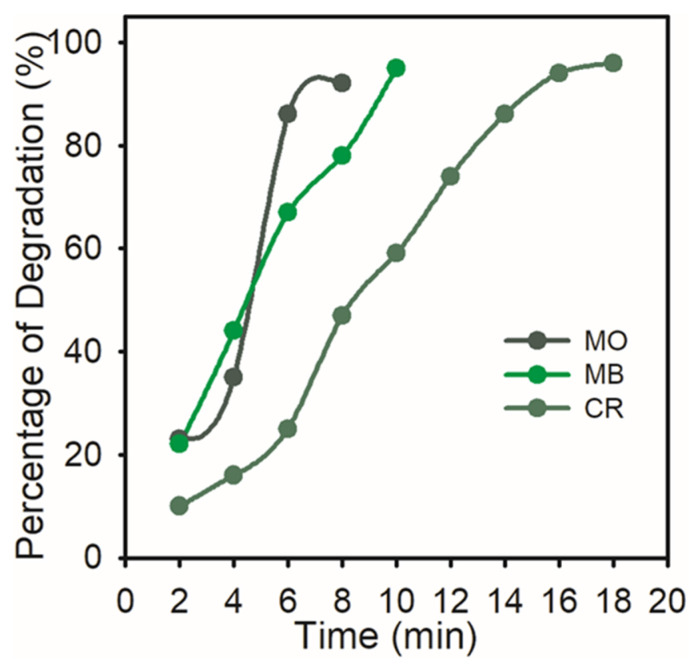
Percent degradation of organic dyes (MO, MB, and CR) with time by AgNPs.

Figure 8 shows the percentage of MO (92%), MB (95%), and CR (96%) that had degraded. Greater catalytic sites and a lower activation energy are provided by the synthesized AgNPs’ high volume-to-surface ratio. Hence, the surface reaction between the reactant and AgNPs may be the cause of the catalytic degradation of organic dyes.

Due to their stability and potent catalytic activity, the biologically produced AgNPs have great potential as catalytic agents for the purification of industrial effluents and wastewater contaminated with organic dyes. Shani Raj et al. have prepared AgNPs by using *Terminalia arjuna* leaf extract and applied them to the reduction of MO, CR, MB, and *p*-NP to *p*-AP. It takes complete degradation over all below 15 min, except for MB, which takes 19 min for complete degradation. Rohini Trivedi’s group has synthesized AgNPs using *Plantago ovata* leaf extract. They found that the AgNPs show good degradation for MB and CR; it takes 20 min of reaction for complete degradation. Very recently, Sushila Singh et al. have prepared AgNPs by using Sapota peel extract and applied them to the reduction of toxic MO dye degradation for 24 min. Compared to previously reported results, this current study’s results are better than those in the literature, as the particle size is less than 100 nm. The results are presented in Table 1.

### 3.6. Catalytic Reduction of Nitrophenol to Aminophenol

**Figure 9:**
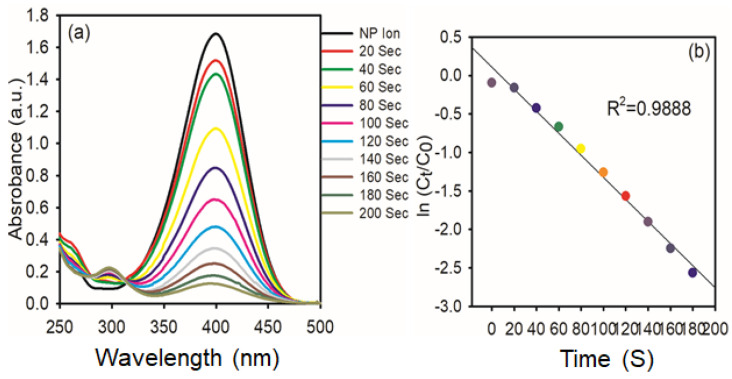
(**a**) Time−dependent UV−vis spectra of the reduction of 4-NP in the presence of AgNPs (0.5 mg); (**b**) Kinetic study of catalytic reduction.

The catalytic reduction of PNP to PAP in the presence of NaBH_4_ is the model reaction used to evaluate the catalytic activity of the synthesized AgNPs. This was accomplished by mixing 1.85 mL (1 mmol) of PNP (the model pollutant), 0.15 mL (0.01 mmol) of NaBH_4_, and 0.5 mg of AgNPs in a UV-cuvette, and the progress of the reaction was measured at predefined intervals. At 317 nm, PNP exhibits an absorption peak. Upon the addition of NaBH_4_, the absorption peak shifts from 317 to 400 nm due to the OH group of PNP deprotonating and forming *p*-nitrophenolate ion, and also the light-yellow color of PNP turns to a deep yellow color [36]. Reports in the literature have revealed that hydride ions of NaBH_4_ and *p*-nitrophenolate ions are repulsive to each other; hence, the metal nanoparticles can enhance the reduction of *p*-NP to *p*-AP due to the fast migration of hydride ions from NaBH_4_. As the reaction progressed, the nitrophenolate ion peak intensity at 400 nm gradually decreased in intensity, while a new peak around 300 nm emerged, indicating the formation of PAP (Figure 9a). Furthermore, the isosbestic points at 281 and 314 nm are confirmed to form PAP without any byproduct, and also the deep yellow color solution turns colorless. The kinetic analysis in Figure 9b involves plotting ln (C_t_/C_0_) against time, where C_t_ and C_0_ are the absorbance values at 400 nm at time zero, respectively. The resulting linear trends and high correlation coefficient (R^2^ = 0.9888) suggest that the reaction follows pseudo-first-order kinetics to PNP under excess NaBH_4_.

**Figure 10:**
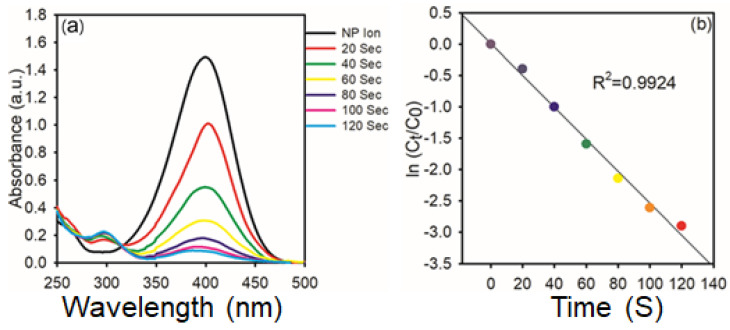
(**a**) Time−dependent UV−vis spectra of the reduction of 4-NP in the presence of AgNPs (1 mg); (**b**) Kinetic study of catalytic reduction.

A similar trend is observed in Figure 10 for the reaction carried out with 1 mg of AgNPs. As shown in Figure 10a, the spectral evolution is more rapid, with complete disappearance of the 400 nm peak within 120 s, and the reduction process’s kinetics were examined using spectrophotometry, as illustrated in Figure 6. It indicates enhanced catalytic activity at higher nanoparticle concentration. The corresponding kinetic plot in Figure 10b again follows a linear relationship with an even higher R^2^ value of 0.9924, reaffirming the pseudo-first-order behavior. The increased rate constant with higher AgNPs dosage highlights the direct dependence of catalytic efficiency on catalyst concentration.

These results collectively demonstrate the high catalytic potential of AgNPs synthesized via the green method. The significant enhancement in the reaction rate with increased catalyst loading is attributed to the greater surface area available for electron transfer processes. Additionally, the clear isobestic points and absence of any side-product peaks in the spectra confirm the selective conversion of PNP to PAP, making this system an efficient and environmentally benign approach for nitroaromatic reduction.

## 4. Conclusions

This study successfully demonstrates that the eco-friendly synthesis of silver nanoparticles (AgNPs) using *L. ovalifolium* flower extract provides a sustainable and efficient alternative to conventional chemical and physical methods. The characterization techniques, including UV–vis spectroscopy, XRD, FTIR, SEM, SEM-EDX, TEM, and DLS, confirm the well-dispersed, spherical AgNPs with a size below 100 nm. These biologically synthesized AgNPs exhibit exceptional catalytic performance in the degradation of toxic dyes such as MO, CR, and MB, achieving degradation efficiencies of 92%, 96%, and 95% respectively, within short time intervals. Furthermore, the AgNPs effectively catalyze the reduction of PNP to PAP in under 4 min, with enhanced reaction rates upon increased catalyst loading. Compared to the existing literature, the current method provides superior catalytic efficiency due to the small particle size and surface area of the AgNPs. Overall, the study emphasizes the potential of *L. ovalifolium* flower extract-derived AgNPs as a cost-effective and environmentally benign solution for water purification and catalytic remediation of hazardous organic pollutants.

## Figures and Tables

**Table 1:** Similar results to those of earlier investigations on the dye-degrading activity of synthesized AgNPs.

Plant Name	Catalyst	Reaction Time (min)			References
		MO	CR	MB	
*Terminalia arjuna*	AgNPs	16	14	19	[32]
*Plantago ovata*	AgNPs	-	18	20	[33]
*Sapota peels*	AgNPs	24	-	-	[34]
*Eulophiaherbacea*	AgNPs	-	30	30	[35]
***L. ovalifolium***	***AgNPs***	***8***	***18***	***10***	***This work***

## Data Availability

The data presented in this study are included in the article.

## References

[1] Al Yahyai W.A.S., Al Isai A.A.S., Alotibi M.F., Reddy B., Al-Abri M., Pejjai B., Devunuri N., Kumar N.S., Al-Fatesh A.S., Osman A.I. (2023). Green synthesis of *Mesona Blumes* gum capped silver nanoparticles and their antioxidant, antibacterial and catalytic studies. Mater. Adv..

[2] Minisy I.M., Salahuddin N.A., Ayad M.M. (2021). Adsorption of methylene blue onto chitosan–montmorillonite/polyaniline nanocomposite. Appl. Clay Sci..

[3] Sherugar P., Padaki M., Naik N.S., George S.D., Murthy D.H. (2022). Biomass-derived versatile activated carbon removes both heavy metals and dye molecules from wastewater with near-unity efficiency: Mechanism and kinetics. Chemosphere.

[4] Hassan M.M., Carr C.M. (2018). A critical review on recent advancements of the removal of reactive dyes from dyehouse effluent by ion-exchange adsorbents. Chemosphere.

[5] Lakkaboyana S.K., Soontarapa K., Asmel N.K., Kumar V., Marella R.K., Yuzir A., Yaacob W.Z.W. (2021). Synthesis and characterization of Cu(OH)_2_-NWs-PVA-AC Nano-composite and its use as an efficient adsorbent for removal of methylene blue. Sci. Rep..

[6] Joga S.B., Korabandi D., Lakkaboyana S.K., Kumar V. (2025). Synthesis of iron nanoparticles on lemon peel carbon dots (LP-CDs@ Fe_3_O_4_) applied in Photo-Catalysis, Antioxidant, Antidiabetic, and Hemolytic activity. Inorg. Chem. Commun..

[7] Azarbani F., Shiravand S. (2020). Green synthesis of silver nanoparticles by *Ferulago macrocarpa* flowers extract and their anti-bacterial, antifungal and toxic effects. Green Chem. Lett. Rev..

[8] Sifonte E.P., Castro-Smirnov F.A., Jimenez A.A.S., Diez H.R.G., Martínez F.G. (2021). Quantum mechanics descriptors in a nano-QSAR model to predict metal oxide nanoparticles toxicity in human keratinous cells. J. Nanoparticle Res..

[9] Sinha A., Behera A. (2022). Nanotechnology in the space industry. Nanotechnology-Based Smart Remote Sensing Networks for Disaster Prevention.

[10] Movahedi R., Razmjoue D., Movahedpour A., Varma R.S., Bahmani M. (2025). Synthesis of Silver Nanoparticles Using *Haplophyllum robustum* Bge. Extract: Antibacterial, Antifungal, and Scolicidal Activity against Echinococcus granulosus Protoscolices. Curr. Nanosci..

[11] Jebril S., Jenana R.K.B., Dridi C. (2020). Green synthesis of silver nanoparticles using Melia azedarach leaf extract and their anti-fungal activities: In vitro and in vivo. Mater. Chem. Phys..

[12] Khatami M., Varma R.S., Zafarnia N., Yaghoobi H., Sarani M., Kumar V.G. (2018). Applications of green synthesized Ag, ZnO and Ag/ZnO nanoparticles for making clinical antimicrobial wound-healing bandages. Sustain. Chem. Pharm..

[13] Mittal A.K., Kaler A., Banerjee U.C. (2012). Free Radical Scavenging and Antioxidant Activity of Silver Nanoparticles Synthesized from Flower Extract of Rhododendron dauricum. Nano Biomed. Eng..

[14] Maddinedi S.B., Mandal B.K., Maddili S.K. (2017). Biofabrication of size controllable silver nanoparticles—A green approach. J. Photochem. Photobiol. B Biol..

[15] Okuda M., Kobayashi Y., Suzuki K., Sonoda K., Kondoh T., Wagawa A., Kondo A., Yoshimura H. (2005). Self-Organized Inorganic Nanoparticle Arrays on Protein Lattices. Nano Lett..

[16] Dai J., Bruening M.L. (2002). Catalytic Nanoparticles Formed by Reduction of Metal Ions in Multilayered Polyelectrolyte Films. Nano Lett..

[17] Roy K., Sarkar C.K., Ghosh C.K. (2014). Photocatalytic activity of biogenic silver nanoparticles synthesized using yeast (*Saccharomyces cerevisiae*) extract. Appl. Nanosci..

[18] Kathiraven T., Sundaramanickam A., Shanmugam N., Balasubramanian T. (2014). Green synthesis of silver nanoparticles using marine algae Caulerpa racemosa and their antibacterial activity against some human pathogens. Appl. Nanosci..

[19] Sajadi S.M., Nasrollahzadeh M., Akbari R. (2019). Cyanation of aryl and heteroaryl aldehydes using in-situ-synthesized Ag na-noparticles in *Crocus sativus* L. Extract. Chem. Sel..

[20] Pawliszak P., Malina D., Sobczak-Kupiec A. (2019). Rhodiola rosea extract mediated green synthesis of silver nanoparticles supported by nanosilica carrier. Mater. Chem. Phys..

[21] Dutta T., Ghosh N.N., Das M., Adhikary R., Mandal V., Chattopadhyay A.P. (2020). Green synthesis of antibacterial and antifungal silver nanoparticles using Citrus limetta peel extract: Experimental and theoretical studies. J. Environ. Chem. Eng..

[22] Rani P., Kumar V., Singh P.P., Matharu A.S., Zhang W., Kim K.-H., Singh J., Rawat M. (2020). Highly stable AgNPs prepared via a novel green approach for catalytic and photocatalytic removal of biological and non-biological pollutants. Environ. Int..

[23] Mahiuddin M., Saha P., Ochiai B. (2020). Green Synthesis and Catalytic Activity of Silver Nanoparticles Based on *Piper chaba* Stem Extracts. Nanomaterials.

[24] Al-Senani G.M., Al-Kadhi N. (2020). The Synthesis and Effect of Silver Nanoparticles on the Adsorption of Cu^2+^ from Aqueous Solutions. Appl. Sci..

[25] Veisi H., Azizi S., Mohammadi P. (2018). Green synthesis of the silver nanoparticles mediated by Thymbra spicata extract and its application as a heterogeneous and recyclable nanocatalyst for catalytic reduction of a variety of dyes in water. J. Clean. Prod..

[26] Bordón D.L., Herrera E., González M.L., Rossi L.I., Aimar M.L., Vázquez A.M., Granados A.M. (2024). Catalytic and biocidal activity of silver and gold nanoparticles obtained by green synthesis using aqueous extracts of glossy privet (*Ligustrum lucidum*) dry fruits. J. Mol. Liq..

[27] Bianca M., Vladislav S., Maria P.-S., Luminita D. (2018). Biosynthesis of Silver Nanoparticles Using *Ligustrum Ovalifolium* Fruits and Their Cytotoxic Effects. Nanomaterials.

[28] Zhao X., Liu J. (2020). Chemical Constituents from the Fruits of Ligustrum lucidum W.T.Aiton and Their Role on the Medicinal Treatment. Nat. Prod. Commun..

[29] Suriyakala G., Sathiyaraj S., Babujanarthanam R., Alarjani K.M., Hussein D.S., Rasheed R.A., Kanimozhi K. (2022). Green synthesis of gold nanoparticles using *Jatropha integerrima* Jacq. flower extract and their antibacterial activity. J. King Saud Univ. Sci..

[30] Somasekhara Reddy M.C., Sivarama Krishna L., Varada Reddy A. (2012). The use of an agricultural waste material, Jujuba seeds for the removal of anionic dye (Congo red) from aqueous medium. J. Hazard. Mater..

[31] Losetty V., Devanesan S., AlSalhi M.S., Velu P.P., Muthupillai D., Kumar K.A., Lakkaboyana S.K. (2024). Green synthesis of silver nanoparticles using Malachra alceifolia (wild okra) for wastewater treatment and biomedical applications with molecular docking approach. Environ. Sci. Pollut. Res..

[32] Raj S., Singh H., Trivedi R., Soni V. (2020). Biogenic synthesis of AgNPs employing *Terminalia arjuna* leaf extract and its efficacy towards catalytic degradation of organic dyes. Sci. Rep..

[33] Githala C.K., Raj S., Dhaka A., Mali S.C., Trivedi R. (2022). Phyto-fabrication of silver nanoparticles and their catalytic dye deg-radation and antifungal efficacy. Front. Chem..

[34] Beniwal A., Singh S., Rani J., Moond M., Kakkar S., Sangwan S., Kumari S. (2024). Waste upcycling of Sapota peels as a green route for the synthesis of silver nanoparticles and their application as catalytic and colorimetric detection of Co^2+^ and Hg^2+^. Nanoscale Res. Lett..

[35] Pawar J.S., Patil R.H. (2019). Green synthesis of silver nanoparticles using *Eulophia herbacea* (Lindl.) tuber extract and evaluation of its biological and catalytic activity. SN Appl. Sci..

[36] Asmare Z.G., Aragaw B.A., Atlabachew M. (2024). Facile Synthesis of Natural Kaolin-Based CuO Catalyst: An Efficient Heterogeneous Catalyst for the Catalytic Reduction of 4-Nitrophenol. ACS Omega.

